# The deubiquitinating enzyme UCHL1 is a favorable prognostic marker in neuroblastoma as it promotes neuronal differentiation

**DOI:** 10.1186/s13046-018-0931-z

**Published:** 2018-10-25

**Authors:** Yuting Gu, Fan Lv, Mingxing Xue, Kai Chen, Cheng Cheng, Xinyuan Ding, Min Jin, Guofeng Xu, Yanyun Zhang, Zhixiang Wu, Leizhen Zheng, Yeming Wu

**Affiliations:** 1 Department of Pediatric Surgery, Xinhua Hospital Affiliated to Shanghai Jiao Tong University School of Medicine, 1665 Kongjiang Road, Shanghai, 200092 China; 2 Department of Stomatology, Renji Hospital Affiliated to Shanghai Jiao Tong University School of Medicine, Shanghai, China; 3 Shanghai Institutes for Biological Sciences, University of Chinese Academy of Science, Chinese Academy of Sciences, Shanghai, China; 4 Division of Pediatric Oncology, Shanghai Institute of Pediatric Research, Shanghai, China; 5 Department of Pharmacy, the Affiliated Suzhou Hospital of Nanjing Medical University, Suzhou, China; 6 Department of Pediatric Surgery, Children’s Hospital of Soochow University, Suzhou, China; 7 Department of Oncology, Xinhua Hospital Affiliated to Shanghai Jiao Tong University School of Medicine, 1665 Kongjiang Road, Shanghai, 200092 China

**Keywords:** Neuroblastoma, UCHL1, Prognosis, Neural differentiation, Proliferation

## Abstract

**Background:**

Neuroblastoma (NB) is the most common pediatric solid tumor that originates from neural crest-derived sympathoadrenal precursor cells that are committed to development of sympathetic nervous system. The well differentiated histological phenotype of NB tumor cells has been reportedly associated with favorable patient outcome. Retinoic acid (RA) can effectively induce NB cell differentiation, thereby being used in the clinic as a treatment agent for inducing the differentiation of high-risk NB. However, the underlying molecular mechanisms of regulating differentiation remain elusive.

**Methods:**

The correlation between clinical characteristics, survival and the deubiquitinating enzyme ubiquitin C-terminal hydrolase 1 (UCHL1) expression were assessed using a neuroblastic tumor tissue microarray, and then validated in three independent patient datasets. The different expression of UCHL1 in ganglioneuroblastoma, ganglioneuroma and NB was detected by immunohistochemistry, mass spectra and immunoblotting analysis, and the correlation between UCHL1 expression and the differentiated histology was analyzed, which was also validated in three independent patient datasets. Furthermore, the roles of UCHL1 in NB cell differentiation and proliferation and the underlying mechanisms were studied by using short hairpin RNA and its inhibitor LDN57444 in vitro.

**Results:**

Based on our neuroblastic tumor tissue microarrays and three independent validation datasets (Oberthuer, Versteeg and Seeger), we identified that UCHL1 served as a prognostic marker for better clinical outcome in NB. We further demonstrated that high UCHL1 expression was associated with NB differentiation, indicated by higher UCHL1 expression in ganglioneuroblastomas/ganglioneuromas and well-differentiated NB than poorly differentiated NB, and the positive correlation between UCHL1 and differentiation markers. As expected, inhibiting UCHL1 by knockdown or LDN57444 could significantly inhibit RA-induced neural differentiation of NB tumor cells, characterized by decreased neurite outgrowth and neural differentiation markers. This effect of UCHL1 was associated with positively regulating RA-induced AKT and ERK1/2 signaling activation. What’s more, knockdown of UCHL1 conferred resistance to RA-induced growth arrest.

**Conclusion:**

Our findings identify a pivotal role of UCHL1 in NB cell differentiation and as a prognostic marker for survival in patients with NB, potentially providing a novel therapeutic target for NB.

**Electronic supplementary material:**

The online version of this article (10.1186/s13046-018-0931-z) contains supplementary material, which is available to authorized users.

## Background

Neuroblastoma (NB) is the most common extracranial childhood solid tumor and the most frequently diagnosed neoplasm during infancy, which representing 8% of childhood cancer diagnoses but 15% of childhood cancer-related deaths [1, 2]. NB originates from neural crest-derived sympathoadrenal precursor cells that are committed to development of the sympathetic nervous system, and is characterized as a heterogeneous group of tumors, ranging from tumors with predominantly poorly differentiated neuroblasts to those largely consisting of fully differentiated sympathetic neurons [3]. The degree of neuronal differentiation in NB reportedly affects patient’s outcome: Patients with NB of differentiating histology have significantly better survival than those with poorly differentiated NB [2, 4, 5]. Retinoic acid (RA), an effective inducer of NB cell differentiation, has been used in the clinic as a differentiation agent for treatment of high-risk NB [6, 7]. However, resistance to RA treatment is observed in NB cell lines, and more than 50% of patients treated with RA develop recurrent NB [7, 8]. Thus far, it is of great clinical value to clarify the molecular mechanisms responsible for NB differentiation, and in order to identify novel therapeutic targets for developing new and more effective therapies.

Ubiquitin C-terminal hydrolase 1 (UCHL1) is a member of ubiquitin carboxy terminal hydrolase family of deubiquitinating enzymes that catalyzes hydrolysis of C-terminal ubiquitin esters and amides to regulate protein degradation [9]. UCHL1 was exclusively expressed in certain organs, such as neurons, diffuse neuroendocrine system and gonads (testis/ovary) [9], and its mutation was associated with neurodegenerative diseases including Parkinson’s disease and Alzheimer’s disease [10, 11]. UCHL1 was also recently studied in cancers, and was considered as either an oncogene or a tumor suppressor gene depending on the tumor types. For example, overexpressed UCHL1 in lymphoma strongly accelerated lymphomagenesis by deregulating PHLPP1 and AKT signaling [12]. In colorectal cancer, UCHL1 acted as an oncogene via activation of the beta-catenin/TCF pathway [13]. The aberrant overexpression of UCHL1 facilitated distant tumor metastases in a HIF-1-dependent manner in breast and lung cancer [14]. However, it has also been found that promoter hypermethylation mediated silencing or low expression of UCHL1 were also found in multiple tumors, including ovarian [15], nasopharyngeal [16], gastric [17], prostate [18], esophageal [19], hepatocellular [20] and breast [21] cancers, indicating UCHL1 as a tumor suppressor. UCHL1 overexpression could induce cell growth arrest in prostate, hepatocellular and breast cancer by stabilizing p53 [16, 20, 21].

UCHL1 has been reported as a marker to detect minimal residual disease in the bone marrow and peripheral blood of NB patients [22–24]. However, the correlation between UCHL1 expression and clinicopathologic features in NB and the effect of UCHL1 on biological behaviors of NB tumor cells are not well understood. As a neuron-specific protein, UCHL1 spatially mediated and enhanced neurogenesis in the embryonic brain by regulating progenitor cell morphology [25]. Satoh et al. also showed that UCHL1 was expressed constitutively in human neural cell lines, where it was upregulated following the induction of neuronal differentiation [26]. In view of these findings, we set out to examine a possible role of UCHL1 in regulating NB cell differentiation.

In the present study, based on our tissue microarray (TMA) and three independent validation datasets (Oberthuer, Versteeg and Seeger), we identified that UCHL1 could serve as a prognostic marker for better clinical outcome in NB. We further demonstrated that high UCHL1 expression was positively associated with NB differentiation, indicating by higher UCHL1 expression in ganglioneuroblastomas (GNB)/ganglioneuromas (GN) and well-differentiated NB than poorly differentiated NB, as well as the positive correlation between UCHL1 expression and differentiation markers. As expected, inhibition of UCHL1 could significantly inhibit RA-induced neural differentiation of NB tumor cells, characterized by decreased neurite outgrowth and neural differentiation markers. This effect of UCHL1 was associated with positively regulating RA-induced AKT and ERK1/2 signaling activation. What’s more, knockdown of UCHL1 also conferred resistance to RA-induced growth arrest. Our findings identify UCHL1 as a critical regulator of NB cell differentiation and as a prognostic marker for survival in patients with NB, and indicate that UCHL1 may serve as a novel therapeutic target for NB.

## Methods

### Patients and tissue specimens

In our study, there were 64 pediatric patients with neuroblastic tumors containing 19 GNB and 38 NB, who were histologically diagnosed in Xinhua hospital affiliated to Shanghai Jiaotong University School of Medicine during October 2012 and February 2015. The recorded data of each patient were analyzed and performed follow-up through phone calls. Each tumor specimen was stored in liquid nitrogen for TMA analysis. 77 pediatric patients with neuroblastic tumors (40 GNB/GN and 37 NB) were histologically diagnosed in Xinhua hospital affiliated to Shanghai Jiaotong University School of Medicine during September 2012 and December 2016. Each tumor specimen was further for high performance liquid chromatography/mass spectra / mass spectra analysis. Protein of 12 of 77 patients was extracted for immunoblotting analysis. All experimental protocols were approved by the Ethics Committee of the Xinhua hospital affiliated to Shanghai Jiaotong University School of Medicine.

### TMA preparation and immunohistochemistry (IHC)

Tissue specimens were separated out a small part and shaped in the special mold for microarray preparation. After fixed in the 4% paraformaldehyde over night, they were trimmed and embedded in paraffin as a planned array. Then, samples were sectioned (5 μm) and attached to poly-L-lysine coated slides. IHC staining was performed using a standard immunoperoxidase staining procedure (primary antibody to UCHL1, 1:400, Cell Signaling Technology, Danvers, Massachusetts, USA). Hematoxylin was used as a counterstain. The tissue sections were viewed independently by two pathologists in a blind fashion. IHC staining was scored on a specialized scale from 0 to 4: 0 represented negative expression, 1 represented weakly positive expression (0–10% positive cells), 2 represented mildly positive expression (10–30% positive cells), 3 represented moderately positive expression (30–50% positive cells), 4 represented strongly positive expression (50–100% positive cells). The scale was determined according to the average number of positive cells in 10 random fields of one slide.

### Validation human datasets

Tissue array analysis results of NB patient tumor samples were obtained from the R2 Genomics Analysis and Visualization Platform (http://r2.amc.nl) using the following publicly available datasets: Oberthuer (ArrayExpress: E-TABM-38) [27], Versteeg (GEO: GSE16476) [28], and Seeger (GEO: GSE3446) [29], which included comprehensive information on the relevant clinical and prognostic factors selected for analysis. Four patients without survival information was not included in the Oberthuer dataset, and the information of age, MYCN status and stage were not contained in the Seeger dataset. For Kaplan-Meier analysis, the best *p* value and corresponding cutoff value was selected according to the R2 Genomics Analysis and Visualization Platform. The Asgharzadeh dataset, which was also obtained from the R2 Genomics Analysis and Visualization Platform, contained 209 neuroblastic tumors with included differentiating histology, and was used for differentiation analysis.

### Cell culture and neural differentiation induction

The human neuroblastoma cell lines, SH-SY5Y and SK-N-BE (2) [30], were obtained from ATCC (Manassas, USA). SH-SY5Y and SK-N-BE (2) cells were maintained in a 1:1 mixture of Eagle’s Minimum Essential Medium and F12 Medium with 10% fetal bovine serum, 1% Penicillin-Streptomycin, 2 mM L-glutamine, 1% non-essential amino acids and 1% Sodium Pyruvate (All from Life Technologies GmbH, Darmstadt, Germany). For neural differentiation assay, *all trans*-RA (Sigma-Aldrich, St Louis, MO) was dissolved in dimethyl sulphoxide (DMSO, Sigma-Aldrich) and 10 mM stock solutions were prepared. SH-SY5Y and SK-N-BE (2) cells were treated with 10 μM RA for 3,5 or 7 days, DMSO was used as negative control. Pictures were taken to monitor neurite outgrowth. The reversible, competitive, active-site directed inhibitor, LDN57444 (Selleckchem, Houston, USA), was used for UCHL1 inhibition [31].

### Lentiviral vector construction

Oligonucleotides with the following nucleotide sequences were used for the cloning of shRNA-encoding sequences into a lentiviral vector plvx-shRNA2 (clontech, Tokyo, Japan): human UCHL1 (sh*UCHL1*), 5’-GATCCCGGGTAGATGACAAGGTGAATCTCGAGATTCACCTTGTCATCTACCCGTTTTTG-3′; Scrambled control (shNC), 5’-GATCCCCTAAGGTTAAGTCGCCCTCGCTCGAGCGAGGGCGACTTAACCTTAGGTTTTTG-3′ (Synthetized by Sangon Biotech). High titer lentiviral stocks were produced, and SH-SY5Y cells were infected with scrambled control lentivirus (shNC) or lentivirus expressing shRNA inhibiting UCHL1 (sh*UCHL1*) according to the manufacturer’s protocol (http://www.clontech.com/). GFP positive cells were selected by flow cytometry sorting and passaged for further study.

### Immunoblotting

Cells were harvested and lysed in the RIPA buffer (Beyotime, Haimen, China) containing PMSF (Beyotime) for 30 min on ice. Lysates were clarified by centrifugation at 15000 g for 30 min. Protein concentration of the supernatant fraction was determined by the Bradford assay. Protein samples were diluted in 4× SDS loading buffer (TaKaRa) and heated to 95 °C for 5 min and fractionated in a 10% or 8% SDS-polyacrylamide gel. Proteins were electroblotted onto a polyvinylidene fluoride and incubated for 1 h in 5% bovine serum albumin in phosphate buffer solution (PBS) or nonfat dry milk dissolved in PBS containing 0.1% Tween-20 (PBST) at room temperature. The blotting membranes were incubated with primary antibodies overnight at 4 °C, extensively washed in PBST, incubated with HRP-conjugated secondary antibody (Cell Signaling Technology) for 1 h at room temperature, and washed again with PBST. The blotting membranes were developed with chemiluminescent reagents (Millipore, Billerica, MA, USA) according to the instructions provide by the manufacturer. Primary antibody to UCHL1, AKT, phospho-AKT (Ser473), ERK1/2, phospho-ERK1/2 (Thr202/Tyr204), p21 and Cyclin D1 were purchased from Cell Signaling Technology, and primary antibody to tyrosine hydroxylase (TH) and growth-associated protein 43 (GAP43) were purchased from Abcam (Cambridge, MA, USA). The blots were quantitative analyzed through the software of Image J (X64, v. 2.1.4).

### Real-time PCR

Total RNA was extracted with TRIzol (Invitrogen) and reverse-transcribed into cDNA with the reverse transcription kit from TakaRa (Tokyo, Japan). The levels of mRNAs were measured by real-time PCR with SYBR Green reagent from Roche (Natley, NJ, USA) and normalized to the level of *GAPDH* mRNA. Primer sequences were as follows: *TH* forward 5′-TCATCACCTGGTCACCAAGTT-3′, reverse 5′-GGTCGCCGTGCCTGTACT-3′*; GAP43* forward 5′-GAGGATGCTGCTGCCAAG-3′, reverse 5′-GGCACTTTCCTTAGGTTTGGT-3′; *TUBB3* forward 5′-CCTGGAACCCGGAACCAT-3′, reverse 5′-AGGCCTGAAGAGATGTCCAAAG-3′; *NPY* forward 5′-TACCCCTCCAAGCCGGACAA-3′, reverse 5′-CATTTTCTGTGCTTTCTCTCAT-3′. All primers were synthesized by Sangon Biotech (Shanghai, China).

### Bromodeoxyuridine (BrdU) assay

Cells with different treatments were incubated with BrdU (BD Biosciences, San Jose, CA, USA) at a final concentration of 10 μM in the cell culture medium for 4 h. The cells were harvested and washed with PBS. After fixation and permeabilization, the cells were treated with 300 μg/ml DNase (Roche). The incorporated BrdU was stained with anti-BrdU-FITC antibody (BD Biosciences) and then analyzed by flow cytometry.

### Cell counting Kit-8 (CCK8) assay

Cell proliferation was monitored using CCK8 (Dojindo, Kumamoto, Japan) according to the manufacturer’s instructions. The cells were seeded onto 96-well plates, and cell proliferation was assessed at the indicated time points by measurement of the absorbance at 450 nm.

### Statistical analyses

Statistical analyses were performed using SPSS version 18.0 software for Windows. The association of UCHL1 expression with clinical pathologic characteristics was analyzed by the chi-square criterion test. Survival analysis was assessed by Kaplan-Meier analysis together with single variable or multivariate Cox analysis. All measurement data are presented as mean ± S.E.M. Statistical significance was evaluated using unpaired nonparametric test. Significance was expressed as: * *P* <  0.05, ** *P* <  0.01, and *** *P* <  0.001.

## Results

### High UCHL1 expression is associated with better clinical outcome in NB

**Fig. 1:**
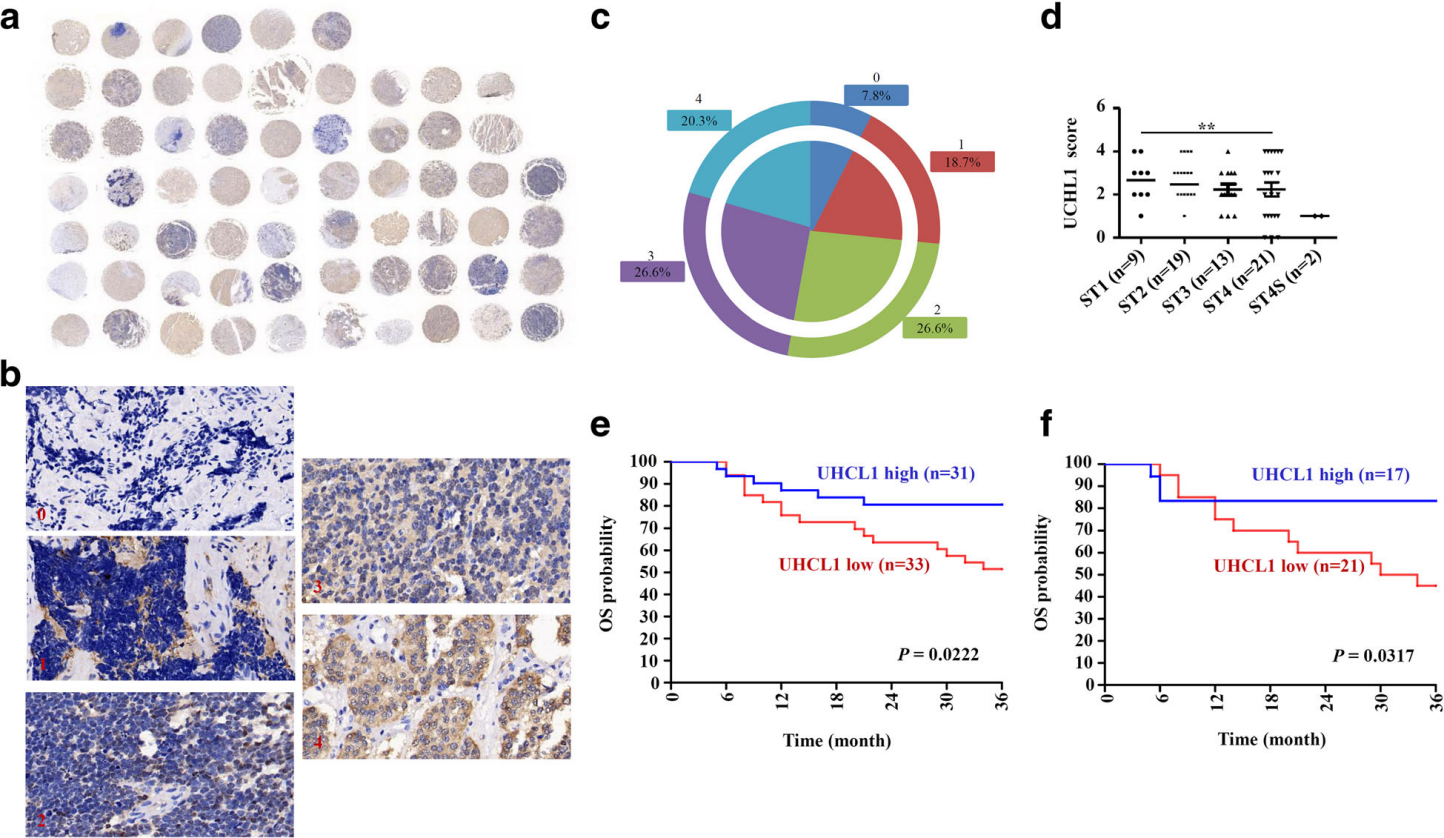
Expression and roles of UCHL1 in TMA of neuroblastic tumors. **a** A general observation of UCHL1 expression in TMA of 64 neuroblastic tumor samples. **b-c** Representative images with five scores (0–4) of IHC staining of UCHL1 expression in neuroblastic tumor samples, and the proportions of five scores were shown. **d** UCHL1 expression in stage (ST) 1-4S tumors. **e** Kaplan-Meier analysis of OS in TMA of 64 neuroblastic tumor samples based on UCHL1 expression with the log-rank test *P* value indicated. **f** Kaplan-Meier analysis of OS of 38 NB samples among the TMA cohort of based on UCHL1 expression with the log-rank test *P* value indicated. Values are shown as mean ± S.E.M. and statistical significance indicated as ** *P* <  0.01

To determine the potential clinic implication of UCHL1 in NB, TMA of neuroblastic tumors obtained from Xinhua Hospital was analyzed by IHC for UCHL1 protein expression. As shown in Fig. 1a-c, UCHL1 was differentially expressed in different samples of neuroblastic tumor patients, according to which patients were classified into UCHL1 high or UCHL1 low based on IHC score. 46.9% of patients demonstrated low UCHL1 expression (IHC score: 0–2), while 53.1% demonstrated high UCHL1 expression (IHC score: 3–4). Then the clinical pathologic characteristics and overall survival (OS) rates were evaluated with respect to UHCL1 expression. There was no significant association of UCHL1 expression with clinicopathologic parameters, such as age at diagnosis, stage, primary site and bone marrow metastasis (Table 1), while UCHL1 expression was higher in tumors of stage 1 than tumors of stage 4 (Fig. 1d). Kaplan-Meier analysis demonstrated that patients with high UCHL1 expression in the TMA cohort showed significantly good OS (3 years OS; UCHL1 high vs. low: 80.6% vs. 51.5%; *P* = 0.0222; Fig. 1e) as compared to the ones with low UCHL1 expression. Further analysis suggested UCHL1 could be scored as a continuous variable retained prognostic significance for OS in single variable analysis (hazard ratio [HR]: 0.698, 95% confidence interval [CI]: 0.506–0.964, *P* = 0.029) and multivariable analysis (HR: 0.738, 95% CI: 0.535–1.018, *P* = 0.064; Tables 2 and 3). In addition, high expression of UCHL1 also showed significantly good OS in NB patients among the TMA cohort (3 years OS; UCHL1 high vs. low: 83.3% vs. 45.0%; *P* = 0.0317; Fig. 1f). Together, these indicates that higher UCHL1 expression in NB patients is associated with better clinical outcome.

**Table 1:** The association between UCHL1 expression with clinical pathologic characteristics in the TMA cohort

Clinical pathologic characteristics	Case No.	UCHL1 expression		*P*
		low	high	
Total cases		33	31	
Gender				
Male	35	17	18	*P* = 0.5989
Female	29	16	13	
Age at diagnosis				
< 18 months	20	11	9	*P* = 0.7106
> 18 months	44	22	22	
stage				
1, 2, 3	41	20	21	*P* = 0.3535
4, 4S	23	13	10	
Primary site				
Retroperitoneum	29	15	14	*P*^a^ = 0.9312
Postmediastinum	8	4	4	
NA	27	14	13	
Bone marrow metastasis				
Positive	7	3	4	*P*^b^ = 0.6054
Negative	26	14	12	
NA	31	16	15	

*NA* Not available. Analysis by chi-square criterion test

^a^Denotes significant difference between retroperitoneum and postmediastinum

^b^Denotes significant difference between positive and negative bone marrow metastasis

**Table 2:** Single variable analyses in the TMA cohort

Covariates	OS	
	HR (95% CI)	*P*
UCHL1 expression	0.698 (0.506–0.964)	0.029

**Table 3:** Multivariable analyses in the TMA cohort

Covariates	OS	
	HR (95% CI)	*P*
UCHL1 expression	0.738 (0.535–1.018)	0.064
Age (< > 18 months)	1.508 (0.548–4.155)	0.427
Stage (4, 4S vs 1, 2, 3)	1.619 (0.674–3.891)	0.281

### UCHL1 is a potential prognostic factor in NB

**Fig. 2:**
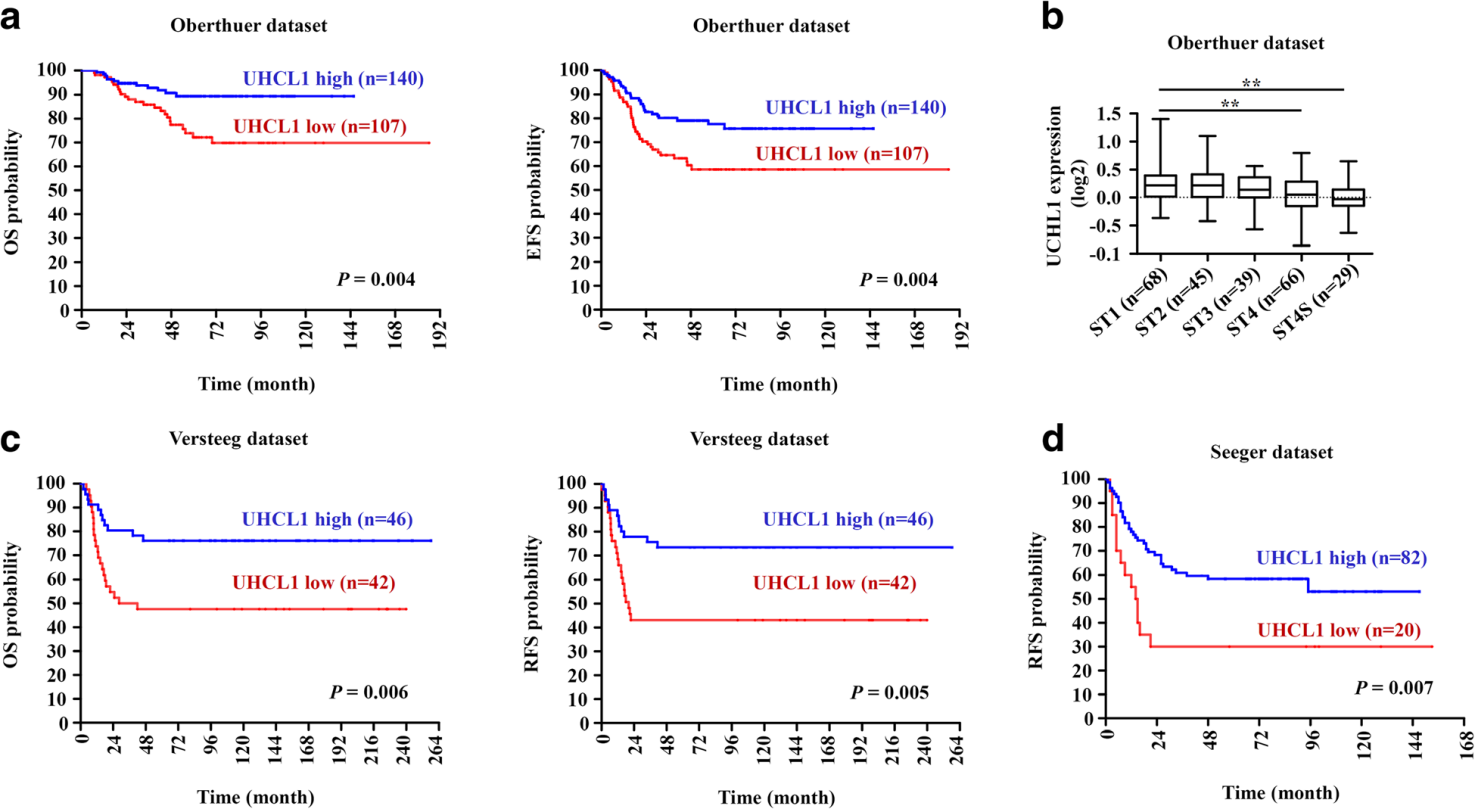
UCHL1’s prognostic value in validation datasets. **a** Kaplan-Meier analysis of OS and EFS for the Oberthuer dataset based on UCHL1 expression with the log-rank test *P* value indicated (*n* = 247). **b** Box plot of UCHL1 expression levels in stage (ST) 1-4S tumors. **c** Kaplan-Meier analysis of OS and EFS for the Versteeg dataset based on UCHL1 expression with the log-rank test *P* value indicated (*n* = 88). **d** Kaplan-Meier analysis of OS and relapse-free survival (RFS) for the Seeger dataset based on UCHL1 expression with the log-rank test *P* value indicated (*n* = 102). Values are shown as mean ± S.E.M. and statistical significance indicated as ** *P* <  0.01

To further evaluate UCHL1 as a potential prognostic factor in NB, we reanalyzed three independent patient datasets (Oberthuer, Versteeg and Seeger) to examine the correlation between the mRNA expression of UCHL1 and the survival rate in NB. In the Oberthuer dataset, patients with high UCHL1 mRNA levels showed better OS (15-year OS; UCHL1 high vs. low: 91.4% vs. 77.6%; *P* = 0.004) and event-free survival (EFS; 15-year EFS; UCHL1 high vs. low: 79.3% vs. 62.6%; *P* = 0.004) (Fig. 2a). In multivariable analysis, increment in every unit of UCHL1 mRNA correlated with a decrease in HR for OS (HR: 0.338, 95% CI: 0.116–0.980, *P* = 0.046) and EFS (HR: 0.476, 95% CI: 0.228–0.994, *P* = 0.048) independent of established risk markers (MYCN status, tumor stage and patient age at diagnosis; Table 4). Moreover, decreased UCHL1 expression correlated significantly with advanced tumor stages (Fig. 2b). By Kaplan-Meier analysis and multivariable analysis, we also confirmed that high UCHL1 expression is prognostic for favorable outcome in the Versteeg and Seeger dataset (Fig. 2c and d; Tables 5 and 6). Taken together, our analyses of three independent microarray datasets also indicate that UCHL1 is a potential prognostic marker in NB.

**Table 4:** Multivariable analyses in the Oberthuer dataset

Subject dataset	Covariates	OS		EFS	
		HR (95% CI)	*P*	HR (95% CI)	*P*
Oberthuer dataset^a^					
UCHL1 expression		0.338 (0.116–0.980)	0.046	0.476 (0.228–0.994)	0.048
Age (< > 18 months)		5.420 (2.249–13.062)	< 0.001	2.292 (1.340–3.919)	0.002
MYCN status (+/− amplification)		5.413 (2.706–10.827)	< 0.001	2.417 (1.443–4.048)	0.001
Stage (4, 4S vs 1, 2, 3)		3.858 (1.732–8.595)	0.001	2.218 (1.252–3.930)	0.006

^a^Four patients without survival information were not included in the Oberthuer dataset

**Table 5:** Multivariable analyses in the Versteeg dataset

Subject dataset	Covariates	OS		RFS	
		HR (95% CI)	*P*	HR (95% CI)	*P*
Versteeg dataset					
	UCHL1 expression	0.402 (0.192–0.842)	0.016	0.441 (0.216–0.899)	0.024
	Age (< > 18 months)	17.097 (4.87–60.018)	< 0.001	6.525 (2.498–17.004)	< 0.001
	MYCN status (+/− amplification)	1.607 (0.769–3.357)	0.207	1.846 (0.625–5.447)	0.267
	Stage (4, 4S vs 1, 2, 3)	1.873 (0.58–6.042)	0.294	1.713 (0.830–3.534)	0.145

**Table 6:** Single variable analyses in Seeger dataset

Subject dataset	Covariates	RFS	
		HR (95% Cl)	*P*
Seeger dataset^a^			
	UCHL1 expression	0.627(0.399–0.985)	0.043

^a^The Seeger dataset contains no information of age, MYCN status and stage

### High UCHL1 expression is associated with NB differentiation

**Fig. 3:**
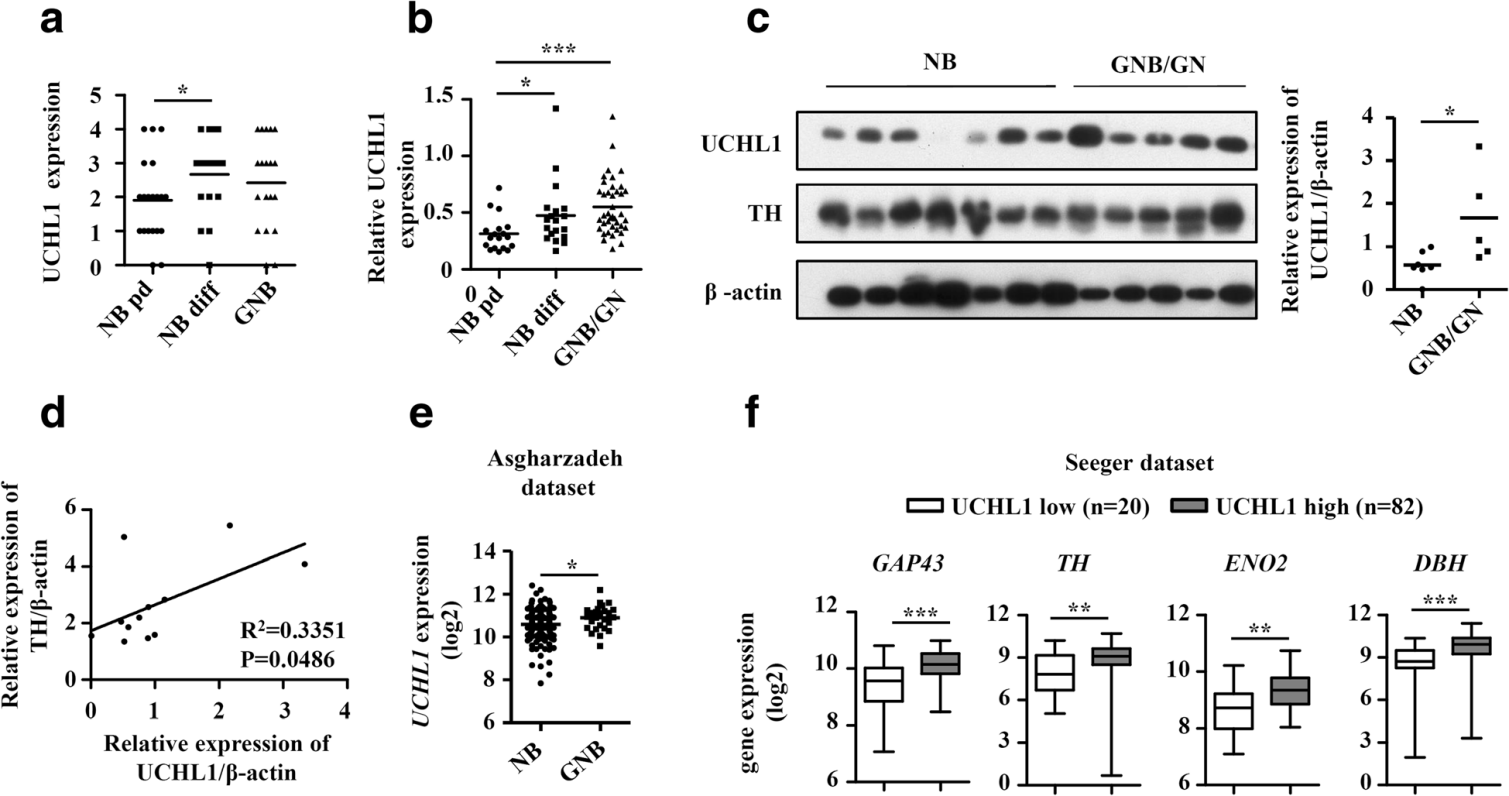
High UCHL1 expression is associated with NB differentiation. **a** IHC score of UCHL1 expression of poorly differentiated NB (NB pd., *n* = 20), well-differentiated NB (NB diff, *n* = 18) and GNB (*n* = 19) tumor samples in the TMA cohort. **b** Mass spectra analysis of tumor tissue extraction of poorly differentiated NB (NB pd, n = 18), well-differentiated neuroblastomas (NB diff, n = 19) and GNB/GN (*n* = 40) patients. UCHL1 was identified and quantified. **c-d** Protein of tumor tissues from NB (*n* = 7) and GNB/GN (*n* = 5) patients was extracted, and UCHL1 and TH protein were measured by immunoblotting analysis. The densitometry of the bands was quantified using ImageJ software, and the correlation between TH and UCHL1 was analyzed. **e** Dot plot of *UCHL1* mRNA expression levels in tumor of NB (*n* = 29) and GNB (*n* = 180) from the Asgharzadeh Dataset. **f** Box plots of individual gene expression levels of *GAP43*, *TH*, *ENO2* and *DBH* in tumors of UCHL1 high and low groups (Seeger dataset) using the UCHL1 expression cutoff value. Values are shown as mean ± S.E.M. and statistical significance indicated as * *P* <  0.05, ** *P* < 0.01 and *** *P* < 0.001

A critical role of UCHL1 in neurogenesis in the embryonic brain has been reported [25], we then analyzed the association of UCHL1 with NB tumor differentiation in the TMA cohort. In TMA samples, 10 of 19 GNB samples and 12 of 18 well-differentiated NB samples demonstrated high UCHL1 expression, compared to 5 of 20 poorly differentiated NB samples (Table 7). Compared with poorly differentiated NB, well-differentiated NB and GNB had higher expression of UCHL1 (Fig. 3a). We further explored UCHL1 expression from another set of samples analyzed by mass spectrometry quantitative proteomics, which comprised of 37 NB and 40 GNB/GN (detail data were not shown). UCHL1 expression was significantly higher in GNB/GN and well-differentiated NB than poorly differentiated NB (Fig. 3b). Immunoblotting analysis also showed that the protein expression of UCHL1 was higher in GNB/GN than NB. Furthermore, UCHL1 expression was positively correlated with the expression of TH, a known differentiation marker in NB (Fig. 3c and d). Similar results were obtained in an independent dataset (Asgharzadeh dataset) of 209 neuroblastic tumors when these data were reanalyzed for UCHL1 mRNA (Fig. 3e). To further confirm the association between UCHL1 and NB differentiation, we investigated the correlation of UCHL1 expression levels with differentiation states in primary NB from the Seeger microarray dataset. When the tumors were separated into UCHL1 high and low expression groups, tumors of the high UCHL1 group expressed significantly higher mRNA levels of differentiation markers such as GAP43, TH, neuronal enolase 2 (ENO2) and dopamine-b hydroxylase (DBH) (Fig. 3f). Similar results could be found in the Oberthuer and Versteeg datasets (Additional file 1: Figure S1). Taken together, our findings illustrate an association of high UCHL1 expression with NB differentiation. We went on to examine how UHCL1 affects NB cell behavior and to explore its signaling functions.

**Table 7:** Low UCHL1 expression is associated with poorly differentiated tumors in the TMA cohort

Clinical pathologic characteristics	Case No.	UCHL1 expression		*P*
		low	high	
Total cases		30	27	
Differentiation				
GNB	19	9	10	*P*^a^ = 0.0315
NB diff	18	6	12	*P*^b^ = 0.0762
NB pd	20	15	5	*P*^c^ = 0.0359

NB diff: well-differentiated NB, NB *pd* poorly differentiated NB

Analysis by chi-square criterion test

^a^Denotes significant difference among the three groups

^b^Denotes significant difference between GNB and poorly differentiated NB

^c^Denotes significant difference between well-differentiated NB and poorly differentiated NB

### UCHL1 promotes neuronal differentiation of NB cells

**Fig. 4:**
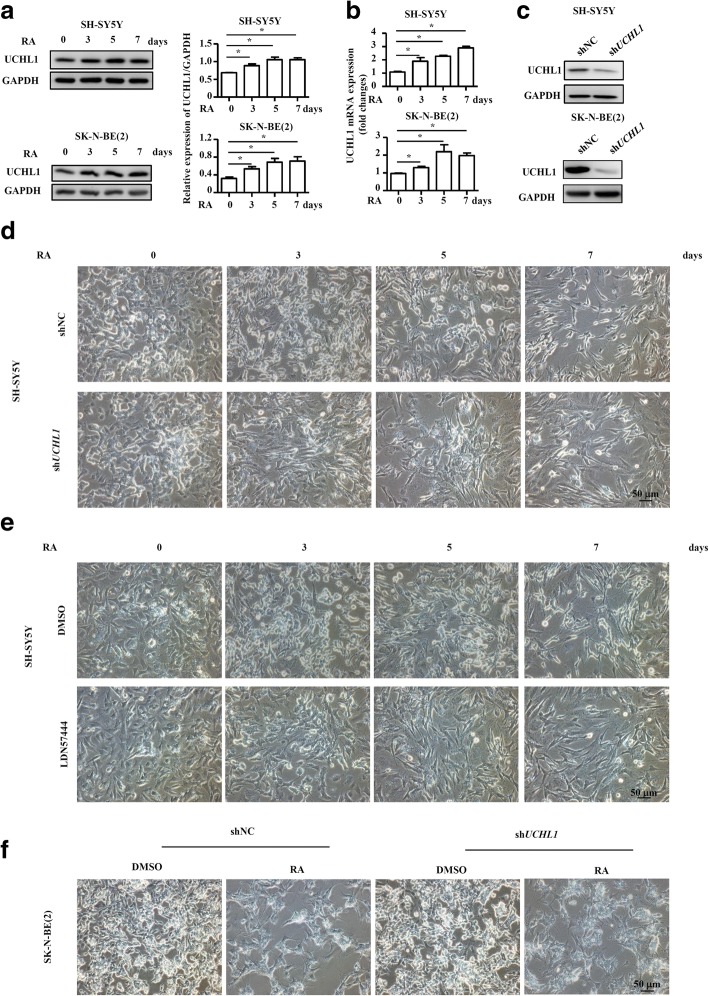
UCHL1 promotes RA-induced neurite outgrowth of NB cells. **a**-**b** SH-SY5Y and SK-N-BE (2) cells were treated with DMSO or RA (10 μM) for 3, 5 and 7 days, protein and mRNA were collected. UCHL1 expression was determined at the protein and mRNA levels by immunoblotting analysis and quantitative real-time PCR. **c** SH-SY5Y and SK-N-BE (2) cells were infected with control lentivirus (shNC) or lentivirus expressing shRNA targeting UCHL1 (sh*UCHL1*). UCHL1 expression was measured by immunoblotting analysis. **d** shNC and sh*UCHL1* SH-SY5Y cells were treated with DMSO or RA (10 μM) for 3, 5 and 7 days. The morphology was examined. Scale bars, 50 μm. **e** SH-SY5Y cells were treated with DMSO or RA (10 μM) for 3, 5 and 7 days in the presence of LDN57444 (10 μM) or not. The morphology was examined. Scale bars, 50 μm. **f** shNC and sh*UCHL1* SK-N-BE (2) cells were treated with DMSO or RA (10 μM) for 7 days. The morphology was examined. Scale bars, 50 μm. Values are shown as mean ± S.E.M. and statistical significance indicated as * *P* < 0.05

**Fig. 5:**
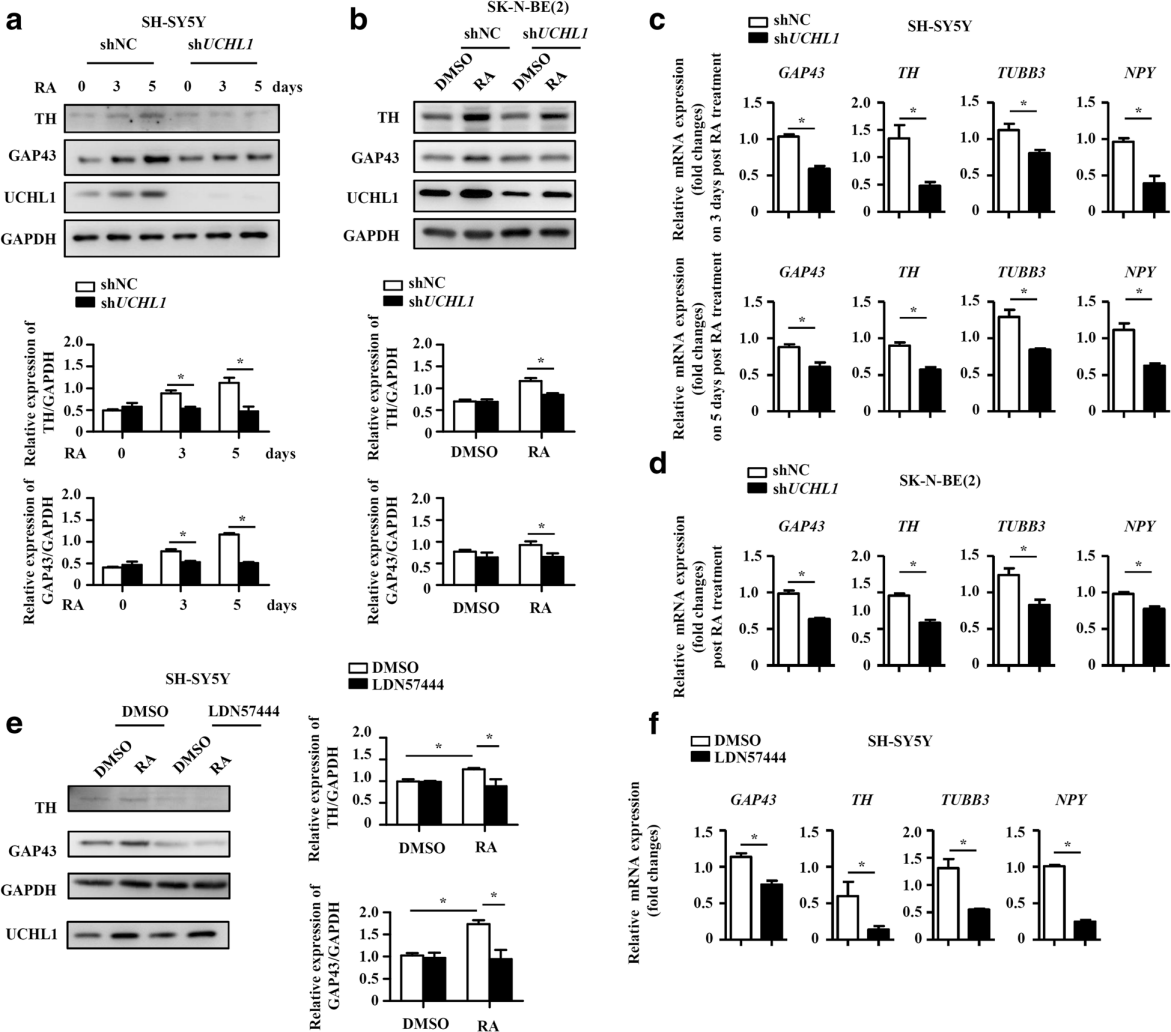
UCHL1 promotes RA-increased neural differentiation markers of NB cells. SH-SY5Y cells were treated with DMSO or RA (10 μM) for 3 and 5 days, and SK-N-BE (2) cells were treated with DMSO or RA (10 μM) for 7 days. **a-b** The protein expression of TH, GAP43 and UCHL1 were measured by immunoblotting analysis at the indicated times and the densitometry of the bands was quantified using ImageJ software. **c-d** The mRNA expression levels of *TH*, *GAP43*, *TUBB3* and *NPY* were measured by quantitative real-time PCR at the indicated times. SH-SY5Y cells were treated with RA (10 μM) for 3 days in the presence of LDN57444 (10 μM) or not. **e** The protein expression of TH, GAP43 and UCHL1 were measured by immunoblotting analysis and the densitometry of the bands was quantified using ImageJ software. **f** The mRNA expression levels of *TH*, *GAP43*, *TUBB3* and *NPY* were measured by quantitative real-time PCR. Values are shown as mean ± S.E.M. and statistical significance indicated as * *P* < 0.05

To gain insight into the role of UCHL1 in neuronal differentiation of NB cells, we used RA, a differentiation agent for clinical treatment of high-risk NB after terminating the chemotherapy, to induce neural differentiation of the NB cell lines, SH-SY5Y and SK-N-BE (2). Results from Real-time PCR and immunoblotting analysis confirmed that UCHL1 was upregulated in SH-SY5Y and SK-N-BE (2) cells upon RA administration (Fig. 4a and b). To evaluate UCHL1 regulating neuronal differentiation, UCHL1 expression was reduced in SH-SY5Y and SK-N-BE (2) cells using a lentivirus-expressing shRNA specific to *UCHL1* (named sh*UCHL1*), and SH-SY5Y and SK-N-BE (2) cells infected with a lentivirus-expressing scrambled shRNA (named shNC) were used as controls (Fig. 4c and Additional file 2: Figure S2). As compared with shNC cells, RA-induced neurite outgrowth was inhibited in sh*UCHL1* SH-SY5Y (Fig. 4d). The reversible, competitive, active-site directed inhibitor of UCHL1, LDN57444, was next used to examine the regulation of UCHL1 on neuronal differentiation. Compared with DMSO-pretreated SH-SY5Y cells, LDN57444-pretreated SH-SY5Y cells had poor capacity of RA-induced neuronal differentiation (Fig. 4e). What’s more, similar phenomenon was also exhibited in SK-N-BE (2) cells (Fig. 4f). To further confirm the function of UCHL1 on RA-induced neuronal differentiation, we detected several established neural differentiation relevant proteins or genes. As shown in Fig. 5a and b, the inhibited neurite outgrowth in sh*UCHL1* SH-SY5Y and SK-N-BE (2) cells was accompanied by downregulation of expression of neuron-specific protein, TH and GAP43. In addition, the established neural differentiation relevant genes, including *TH*, *GAP43*, *TUBB3*, and *NPY were* down-regulated in RA-treated SH-SY5Y and SK-N-BE (2) cells after UCHL1 knockdown in comparison to the controls (Fig. 5c and d). Similar changes of neural differentiation relevant proteins or genes were also detected in LDN57444-pretreated SH-SY5Y cells (Fig. 5e and f). These results together suggest that UCHL1 promotes neuronal differentiation of NB cells.

**Fig. 6:**
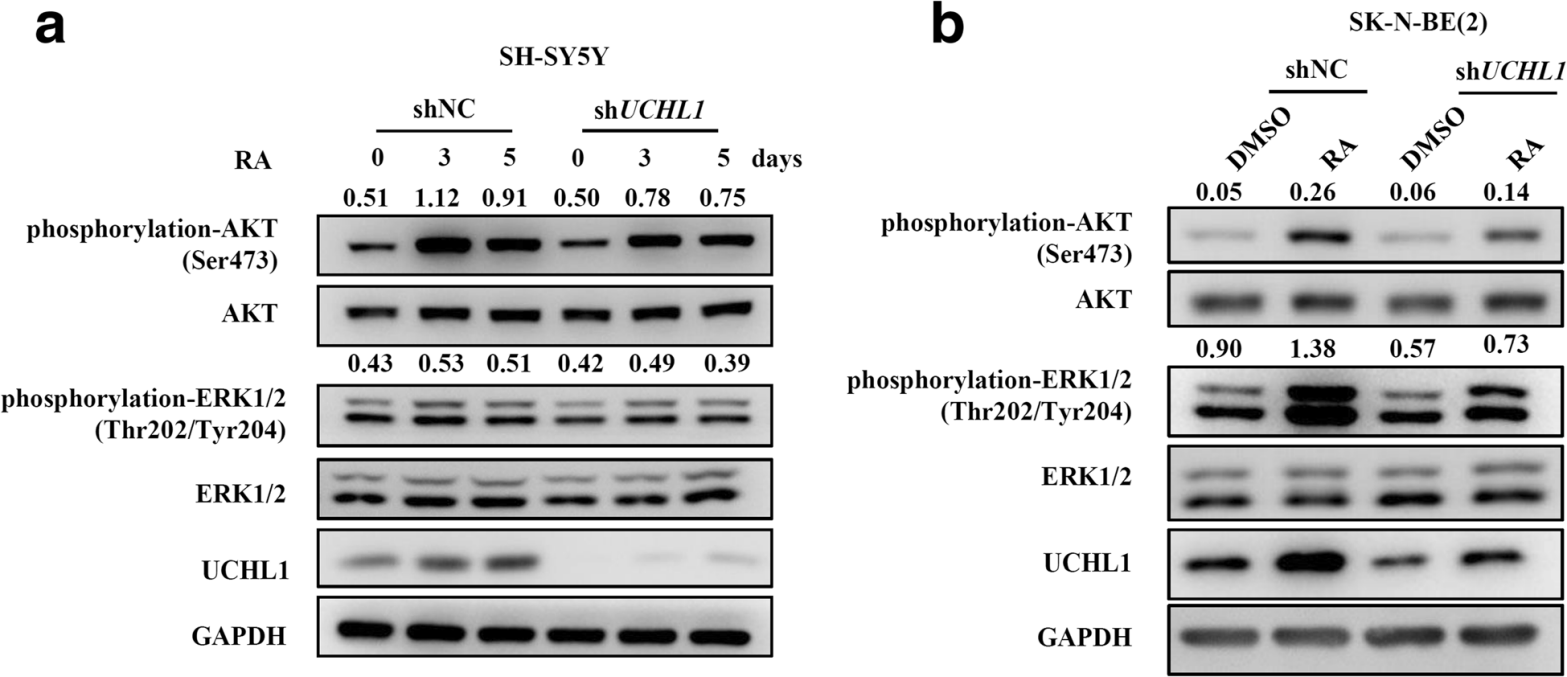
Inhibition of differentiation by UCHL1 dysregulation is associated with AKT and ERK1/2 signaling. **a** shNC and sh*UCHL1* SH-SY5Y cells were treated with DMSO or RA (10 μM) for 3 and 5 days. **b** shNC and sh*UCHL1* SK-N-BE (2) cells were treated with DMSO or RA (10 μM) for 7 days. The protein expression of phospho-AKT (Ser473), AKT, phospho-ERK1/2 (Thr202/Tyr204), ERK1/2 and UCHL1 were measured by immunoblotting analysis in the indicated time. The densitometry of the bands was quantified using ImageJ software, and AKT and ERK1/2 were used as controls, respectively

To explore the potential molecular mechanisms by which UCHL1 promoted neuronal differentiation, we performed immunoblotting analysis to determine the activities of signaling pathways including AKT and ERK1/2, in cells. These signaling pathways are known as downstream of RA signaling and critical for RA-induced differentiation [32, 33]. As shown in Fig. 6a and b, UCHL1 knockdown dramatically inhibited the phosphorylation of AKT at Ser473 and Erk1/2 at Thr202/Tyr204 after RA administration in both SH-SY5Y and SK-N-BE (2) cells. These results suggest that the effect of UCHL1 inhibition on RA-induced neuronal differentiation is mediated by repression of AKT and ERK1/2 activities.

### UCHL1 promotes RA-repressed proliferation of NB cells

**Fig. 7:**
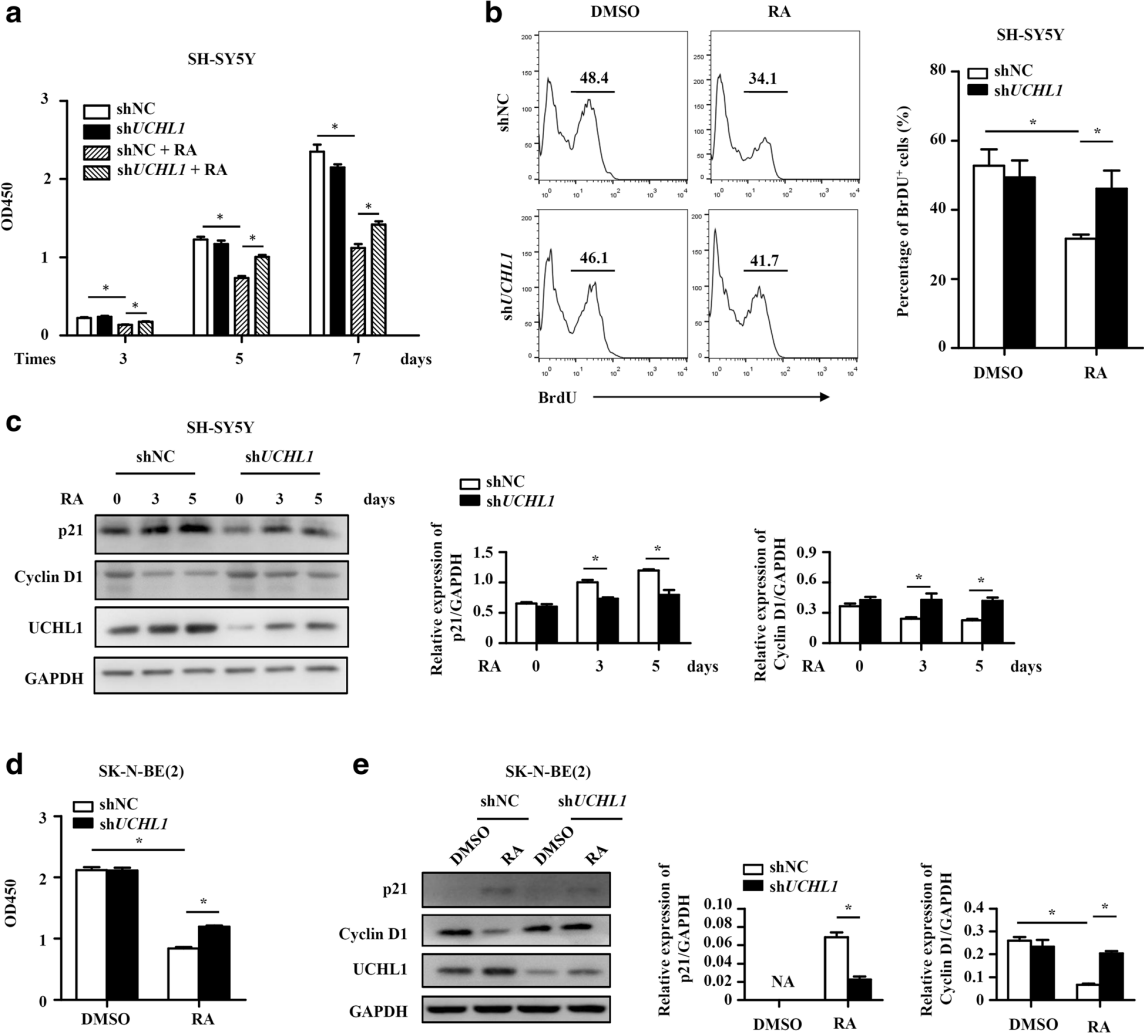
UCHL1 promotes RA-induced proliferation inhibition of NB cells. shNC and sh*UCHL1* SH-SY5Y cells were treated with DMSO or RA (10 μM) for 3, 5 or 7 days. **a** BrdU assay showed the proliferation of shNC and sh*UCHL1* SH-SY5Y cells after DMSO or RA treatment for 3 days, and the percentage of BrdU^+^ SH-SY5Y cells was analyzed by flow cytometry. **b** CCK8 assay showed the proliferation of shNC and sh*UCHL1* SH-SY5Y cells at the indicated times. **c** The protein expression of p21, cyclin D1 and UCHL1 were measured by immunoblotting analysis at the indicated times, and the densitometry of the bands was quantified using ImageJ software. shNC and sh*UCHL1* SK-N-BE (2) cells were treated with DMSO or RA (10 μM) for 7 days. **d** CCK8 assay showed the proliferation of shNC and sh*UCHL1* SK-N-BE (2) cells. **e** The protein expression of p21, cyclin D1 and UCHL1 were measured by immunoblotting analysis, and the densitometry of the bands was quantified using ImageJ software. Values are shown as mean ± S.E.M. and statistical significance indicated as * *P* < 0.05

UCHL1 could either positively or negatively regulate cell proliferation of cancer cells [34]. To further characterize the potential function of UCHL1 in NB, we examined whether UCHL1-regulated cell differentiation was coupled with regulation of NB cell proliferation. BrdU and CCK8 assays showed that there was no significant difference of the proliferation between shNC and sh*UCHL1* SH-SY5Y cells (Fig. 7a and b). In addition to promoting NB neural differentiation, RA also could induce NB cell growth arrest [6, 7]. Here we found that UCHL1 knockdown reversed RA-induced cell growth arrest of SH-SY5Y cells, characterized by increased percentage of BrdU^+^ cells and cell viability in sh*UCHL1* SH-SY5Y cells in the presence of RA (Fig. 7a and b). Next, we tested the levels of the cell cycle regulators p21 and cyclin D1, which regulated cell proliferation. As shown in Fig. 7c, as compared with shNC SH-SY5Y cells, RA-upregulated cell cycle-dependent kinase inhibitor p21 level and RA-downregulated cyclin D1 level was inhibited in sh*UCHL1* SH-SY5Y cells, consistent with the effect of UCHL1 on RA-induced inhibition of cell proliferation. Besides, we also examined the effects of UCHL1 on RA-repressed proliferation of SK-N-BE (2) cells, and showed the similar phenomenon in Fig. 7d and e. These results indicate that UCHL1 also promotes RA-repressed proliferation of NB cells.

## Discussion

NB is a pediatric solid tumor that originates from embryonal neural crest cells committed to the development of the sympathetic nervous system. In most cases, NB presents as a tumor consisting of poorly differentiated small round blue cells, which may differentiate into more benign tumor phenotypes (i.e., GNB/GN) after either initiating spontaneously regression or following cytotoxic treatment. The ubiquitin ligases and deubiquitinating enzymes-mediated ubiquitination and deubiquitination have been reported involved regulating various biological behaviors of this malignancy, including developmental differentiation processes [32, 35]. However, the underlying molecular mechanisms of differentiation remain elusive. Our present study provides several lines of evidence for the hypothesis that the deubiquitinating enzyme UCHL1 is a critical regulator of NB differentiation.

UCHL1 was reported as a marker to detect minimal residual disease in the bone marrow and peripheral blood of NB patients by reverse transcription PCRs, while UCHL1 mRNA was very low in the study of Corrias MV et al. [22–24]. However, UCHL1 expression in NB tumor cells and the association between UCHL1 expression with clinicopathologic features and survival of NB patients are not well understood. Our clinical observations showed that there were no significant association of UCHL1 expression with clinicopathologic parameters, such as age at diagnosis, stage, primary site and bone marrow metastasis. However, patients with high expression of UCHL1 in the TMA cohort of neuroblastic tumors held significantly good OS. Particularly, UCHL1 could score as a continuous variable retained prognostic significance for OS in single variable analysis and multivariable analysis. Besides, high UCHL1 expressed NB patients among the TMA cohort also showed significantly good OS, indicating the strongly association between high UCHL1 expression levels and beneficial outcome in NB. Similar observation could be found in three independent validation datasets (Oberthuer, Versteeg and Seeger).

Recent studies have found that UCHL1 plays important roles in cell differentiation. For example, Gao H et al. demonstrated that UCHL1 played a role in myogenesis by inhibiting differentiation [36], while Sakura et al found that UCHL1 could regulate the morphology of neural progenitor cells and modulate their differentiation thereby enhancing neurogenesis in the embryonic brain [25]. In this study, we found that UCHL1 expression was significantly higher in GNB/GN and well-differentiated NB than poorly differentiated NB, and UCHL1 expression was positively correlated with the known neural differentiation markers. What’s more, UCHL1 expression was increased during RA-induced neural differentiation of SH-SY5Y and SK-N-BE (2) cells, which was consistent with the conclusion of Satoh et al [26]. Intervening UCHL1 by shRNA as well as the small specific molecular inhibitor could significantly inhibit RA-induced neural differentiation in NB tumor cells, characterized by decreased neurite outgrowth and neural differentiation markers. Together, these results suggested that UCHL1 had a vital role in NB neuronal differentiation, at least by the means of synergistic effect with RA.

The contrary roles of UCHL1 have been reported in different types of cancers, associated with poor or better survival [34]. In several tumor types (e.g. esophageal, hepatocellular and breast cancers), low UCHL1 expression has reportedly been attributed to promoter hypermethylation. UCHL1 promoter hypermethylation is prognostic for poor clinical outcome, and DNA methyltransferase inhibitor could induce UCHL1 expression for therapeutic effects [19–21]. In NB, gene repression by genomic methylation has been shown to be involved in several putative tumor suppressor genes [37, 38], such as the tumor suppressor gene *TFAP2B*, promoter methylation of which is an independent prognostic factor for NB [39]. Besides, Fakhera et al. found that DNA methyltransferase inhibitor could rescue *TFAP2B* in IMR-32 NB cells thereby promoting their differentiation. Thus, it is of great interest whether UCHL1 expression is regulated by promoter hypermethylation and whether this regulation is involved in NB.

Among several pathways that have been identified for the RA-induced NB differentiation, AKT and ERK1/2 are involved by regulating RXR transcription activity [32, 33]. AKT and ERK1/2 was positively regulated by UCHL1 in B cells [40], gastric cancer cells [17] and breast cancer cells [41]. Here, we demonstrated similar mechanisms in NB cells that UCHL1 inhibition dramatically inhibited the phosphorylation of AKT at Ser473 and ERK1/2 at Thr202/Tyr204 after RA administration, indicating that the effect of UCHL1 inhibition on RA-induced neuronal differentiation was associated with repression of AKT and ERK1/2 activities.

UCHL1 has been reported positively or negatively regulating cell proliferation [34]. UCHL1 could promote proliferation of B-cell lymphoma and gastric cancer cells [40, 42]; while others demonstrated that UCHL1 induced cell cycle arrest and inhibited tumor cell proliferation in breast, ovarian and hepatocellular cancer [15, 20, 21]. Besides, Ichikawa et al found that UCHL1 could negatively regulate TNFα-mediated vascular smooth muscle cell proliferation [43]. In the present study, we found that UCHL1 knockdown had no effects on SH-SY5Y and SK-N-BE (2) cells. However, UCHL1 knockdown reversed RA-induced cell growth arrest of SH-SY5Y and SK-N-BE (2) cells, accompanied with reversal of RA-upregulated cell cycle-dependent kinase inhibitor p21 level and RA-downregulated cyclin D1. These indicated that UCHL1-promoted cell differentiation in the presence of RA was coupled with regulation of cell proliferation.

## Conclusion

In summary, the current study showed that UCHL1 could serve as a prognostic marker indicating a favorable clinical outcome in NB. UCHL1 expression was higher in GNB/GN and well-differentiated NB than poorly differentiated NB, and UCHL1 expression was strongly associated with differentiation markers, indicating the positive correlation between UCHL1 expression and NB differentiation. As expected, inhibition of UCHL1 could significantly inhibit RA-induced neural differentiation of NB tumor cells. And this effect of UCHL1 was associated with positively regulating RA-induced AKT and ERK1/2 signaling activation. What’s more, loss function of UCHL1 also could confer the resistance to RA-induced growth arrest. Therefore, our study provides insights into a critical regulator of UCHL1 for NB cell differentiation and as a prognostic marker for survival in patients with NB, and potentially provides novel therapeutic targets for NB.

## Additional files

Additional file 1: **Figure S1.** a**-**b Box plots of individual gene expression levels of *GAP43*, *TH*, *ENO2* and *DBH* in tumors of UCHL1 high and low groups in the Oberthuer and Versteeg datasets using the UCHL1 expression cutoff value. Values are shown as mean ± S.E.M. and statistical significance indicated as *** *P* <  0.001. (TIF 658 kb).

Additional file 2: **Figure S2.** SH-SY5Y and SK-N-BE (2) cells were infected with control lentivirus (shNC) or lentivirus expressing shRNA targeting UCHL1 (sh*UCHL1*). a UCHL1 expression was measured by immunoblotting analysis and the densitometry of the bands was quantified using ImageJ software. b The mRNA expression of UCHL1 was measured by quantitative real-time PCR. Values are shown as mean ± S.E.M. and statistical significance indicated as * *P* <  0.05. (TIF 680 kb).

## Abbreviations

BrdU: Bromodeoxyuridine
CCK8: Cell counting kit-8
CI: Confidence interval
DBH: Dopamine-b hydroxylase
DMSO: Dimethyl sulphoxide
EFS: Event-free survival
ENO2: Neuronal enolase 2
GAP43: Growth-associated protein 43
GN: Ganglioneuromas
GNB: Ganglioneuroblastomas
HR: Hazard ratio
IHC: Immunohistochemistry
NB: Neuroblastoma
OS: Overall survival
PBS: Phosphate buffer solution
PBST: PBS containing 0.1% tween-20
RA: Retinoic acid
RFS: Relapse-free survival
TH: Tyrosine hydroxylase
TMA: Tissue microarray
UCHL1: Ubiquitin C-terminal hydrolase 1
