# Supplementary figures and images for: The deubiquitinating enzyme UCHL1 is a favorable prognostic marker in neuroblastoma as it promotes neuronal differentiation
Source: J Exp Clin Cancer Res. 2018 Oct 25;37:258. doi: 10.1186/s13046-018-0931-z (PMC6203192; doi:10.1186/s13046-018-0931-z)

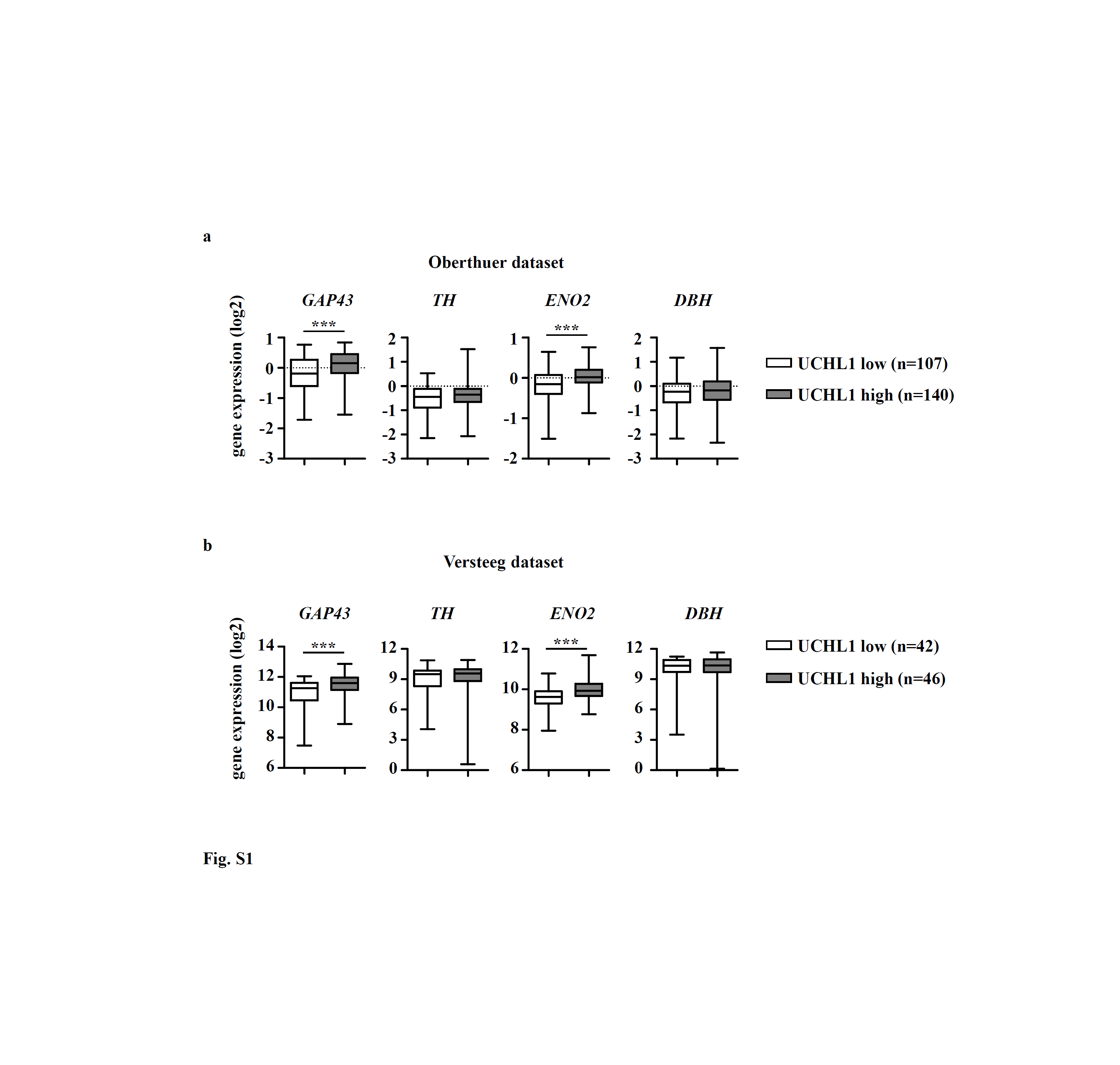

Supplement: Supplementary file 1 — Figure S1. a-b Box plots of individual gene expression levels of GAP43, TH, ENO2 and DBH in tumors of UCHL1 high and low groups in the Oberthuer and Versteeg datasets using the UCHL1 expression cutoff value. Values are shown as mean ± S.E.M. and statistical significance indicated as *** P < 0.001. (TIF 658 kb). [file 13046_2018_931_MOESM1_ESM.tif]

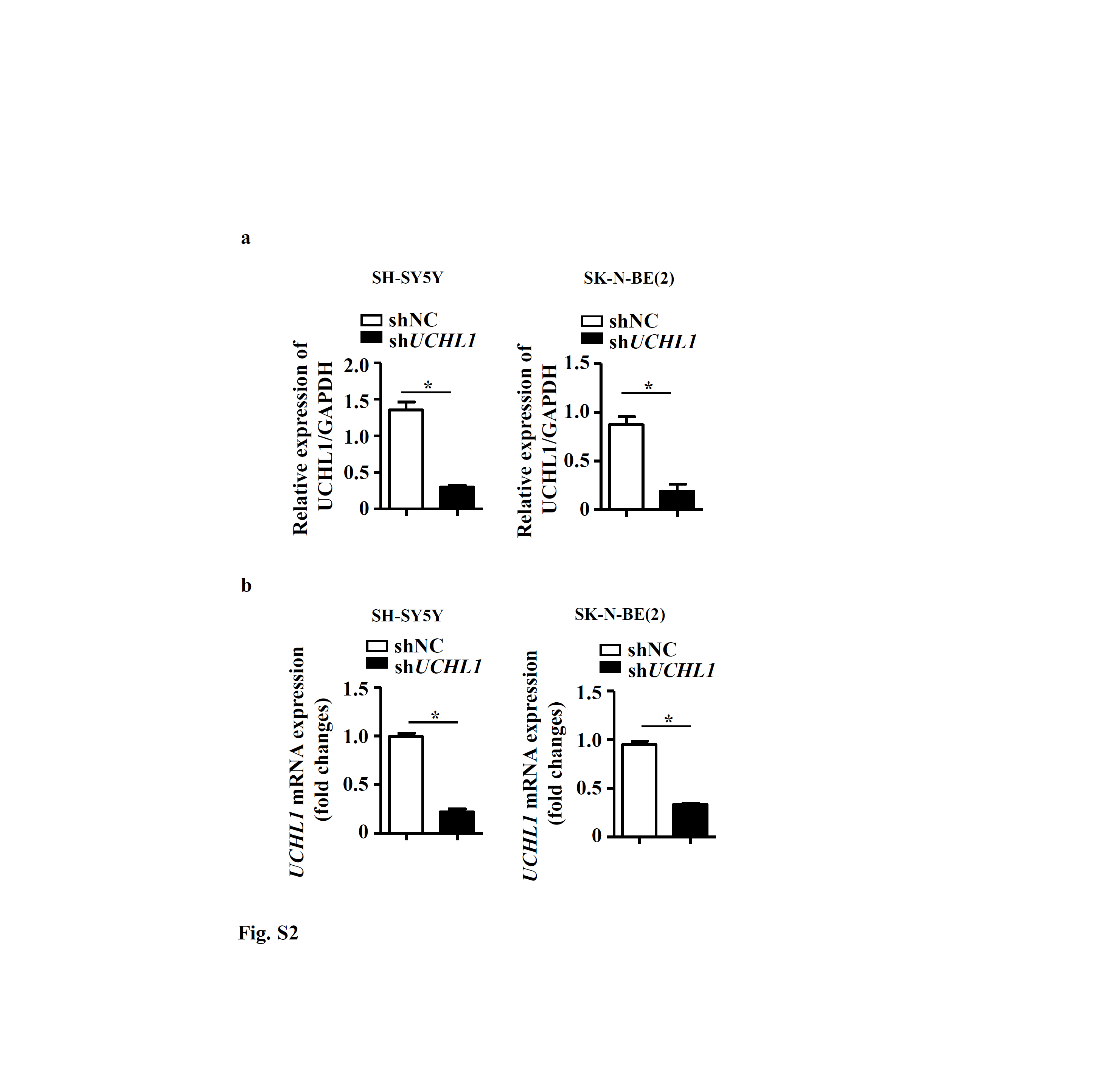

Supplement: Supplementary file 2 — Figure S2. SH-SY5Y and SK-N-BE (2) cells were infected with control lentivirus (shNC) or lentivirus expressing shRNA targeting UCHL1 (shUCHL1). a UCHL1 expression was measured by immunoblotting analysis and the densitometry of the bands was quantified using ImageJ software. b The mRNA expression of UCHL1 was measured by quantitative real-time PCR. Values are shown as mean ± S.E.M. and statistical significance indicated as * P < 0.05. (TIF 680 kb). [file 13046_2018_931_MOESM2_ESM.tif]
